# Mindset as Characteristic Adaptations: Using Response Surface Analysis to Assess Mindset in the Personality System

**DOI:** 10.3389/fpsyg.2021.701510

**Published:** 2021-07-20

**Authors:** Juliette L. Ratchford, Emily G. Williams, Leanne Bishara, Benjamin J. Houltberg, Sarah A. Schnitker

**Affiliations:** ^1^Science of Virtues Laboratory, Department of Psychology and Neuroscience, Baylor University, Waco, TX, United States; ^2^Fuller Theological Seminary, School of Psychology, Pasadena, CA, United States; ^3^Search Institute, Minneapolis, MN, United States

**Keywords:** mindset, personality, characteristic adaptations, well-being, response surface analysis

## Abstract

This study aimed to assess the congruencies and discrepancies between mindset domains in relation to well-being and sought to demonstrate that mindset falls into the characteristic adaptation level of personality. Data (*N* = 618, *M*_*age*_ = 16.07, *SD*_*age*_ = 0.99) from Wave 1 of a longitudinal study on primarily ethnic-minority adolescents were used in response surface analyses to examine the effects of (in)congruence on well-being. The response surface analyses suggested no overall congruence effect between moral and ability mindsets. However, two-thirds of the participants demonstrated differing levels of mindsets, highlighting the domain specificity of mindsets. Results suggest that mindsets are contextual, domain-specific constructs, suiting the characteristic adaptation level of personality. Congruence for moral and ability mindset does not affect adolescent well-being.

## Introduction

In the three decades since Dweck and Leggett (1988) first defined mindsets, or the tendencies that people hold in viewing their capabilities and attributes as more or less malleable, research on the topic has proliferated. Dweck and Leggett (1988) compared two types of mindsets: growth mindset, wherein the person believes that certain attributes (e.g., intelligence) are inherently malleable and can be changed, and fixed mindset, in which the person believes that a certain attribute is an unchangeable, static trait. Individuals vacillate across a spectrum with growth mindset at one pole and fixed mindset at the other (Dweck and Leggett, 1988). Research has largely placed an emphasis on developing interventions to foster growth mindsets in children and adolescents (Dweck and Yeager, 2019). Yet, even with the increasing interest in mindsets, little research has investigated mindset variability across distinct domains.

People hold mindsets across many unique domains including intelligence (e.g., Dweck and Leggett, 1988), morality (e.g., Chiu et al., 1997), ability (e.g., Wang et al., 2018), and emotion (e.g., Tamir et al., 2007). Moral mindsets consider beliefs regarding the nature of the moral self (Chiu et al., 1997), whereas ability mindsets may develop in any performance-based domain including music, art, and sports (Wang et al., 2018). The way people organize their goals within the self-system, regulate their behavior and emotions in the pursuit of goals, and flourish in a given domain is largely dependent on their mindsets, with a more growth mindset typically predicting higher goal achievement (e.g., Dweck and Leggett, 1988; Dupeyrat and Mariné, 2005).

Researchers studying mindsets typically choose a specific domain to investigate that best fits the context of their study, following Dweck (1996) suggestion to adapt items to specific context of the participants (e.g., studying athletic ability in sporting contexts, studying intelligence in academic contexts). Researchers have yet to investigate differences and similarities in mindsets across domains and the way congruencies and discrepancies might impact outcomes. Moreover, little theory or research examines how congruence and incongruence across mindset domains might impact the larger personality system.

### Mindset Within the Personality System

Current research typically investigates specific domains of mindset in relation to expected, contextual outcomes. Such a focus suggests an underlying assumption that mindset domains diverge from one another, but this assumption has yet to be investigated empirically. Most psychological measures do not attend to contextual influences, instead measuring global, wholistic constructs that assess general tendencies across all situations and domains (Furr, 2009). Global measures typically invoke dispositional traits, the broad consistencies across contexts and time (e.g., agreeableness; Big Five). The extant measures of mindsets diverge from this approach, as each is focused on a specific domain (e.g., ability; morality). This approach is best conceptualized at the characteristic adaptation level of personality, which involves contextualized, specific motivational, social-cognitive, and developmental variables (McAdams and Pals, 2006). If empirical research does not support the domain specificity of mindsets, then researchers might consider whether a trait-based conceptualization is better suited than a characteristic adaptation conceptualization.

Mindset is not typically assessed alongside dispositional traits, and the scant existing research suggests that mindsets are typically uncorrelated with the Big Five traits (e.g., Spinath et al., 2003; Burnette and Pollack, 2013). Higher growth intelligence mindset has been related to higher conscientiousness and lower neuroticism (Satchell et al., 2017). However, there is little consensus on the relation between mindsets across multiple domains and dispositional traits. The present study seeks to investigate mindsets for ability and morality in relation to the Big Five traits, which will further clarify relations across the different personality levels and situate mindset within the broader personality system.

The two domains of mindsets of particular interest in the present study are ability and morality. These two domains were selected because of the context of our sample, following Dweck (1996) suggestion to contextualize the mindsets. Our sample consists of adolescents who are mostly engaged in an athletic context (71.9% of sample). In pairing these two domains together, we attempt to tap into how the congruencies between them affect well-being outcomes.

These two domains are often linked with distinct outcomes. Ability mindset is typically associated with outcomes related to self-regulation, achievement, and performance (Burnette and Pollack, 2013), whereas morality mindset is linked to outcomes related to prosociality (Han et al., 2018). These diverging outcomes likely reflect the distinct foci of the two domains. Whereas, ability mindset is focused on people's beliefs about their propensity to be skilled and talented, morality mindset reflects people's beliefs about their innate goodness and moral character of the self and others.

### Do Congruencies and Discrepancies Across Mindset Domains Matter?

Despite numerous studies demonstrating the importance of growth mindset in bolstering and buffering well-being, no current studies explore the effects of (in)congruence across different mindset domains on well-being. However, researchers might expect that being highly growth-minded in one domain while being highly fixed-minded in another could lead to detrimental outcomes. Consider the example of Denise, a high school volleyball athlete who has a growth mindset in morality but a fixed mindset in ability. Denise believes that people are capable of changing their moral character but are unable to change their abilities and talents. In situations where only ability or morality are relevant, these distinct mindsets would not affect well-being, but incoherence may occur in scenarios where moral and ability domains overlap. Suppose Denise was struggling in her abilities to perform well in the previous match, so she cheated to make up for her deficits. Would Denise believe that she can change her behavior in the future, which would preference her growth-minded approach to morality, or will she think she is stuck in this pattern of failure, which would preference her fixed-minded approach to her athletic ability? This resulting dissonance among her mindsets could cause conflicts in the self-system that affect her self-regulation and well-being. Moreover, the quantity or quality of the dissonance Denise experiences might differ from her teammates who have other patterns of mindset convergence and divergence. For example, Rachel has a fixed mindset in morality and a growth mindset in ability, and Alexis, Lauren, and LaTonya have matching ability and moral mindsets—but at low, medium, and high levels of growth, respectively. We endeavor to understand whether and how these combinations of mindsets affect self-regulation and well-being.

Because of the dearth in the literature on (in)congruence across mindset domains, research on related characteristic adaptations is necessary to form preliminary hypotheses. Meta-analytic results demonstrate that growth ability mindset predicts distinctive self-regulatory, goal-oriented processes (e.g., goal setting, goal operating, and goal monitoring), which ultimately lead to goal achievement (Burnette and Pollack, 2013). Thus, examination of (in)congruence among personal goals and strivings, may provide the closest parallels with mindsets to form our hypotheses. Harmony among goals is often associated with positive effects, whereas conflict is correlated with maladaptive outcomes (e.g., Sheldon and Elliot, 1999; Gray et al., 2017). On a theoretical level, we expect that congruencies across mindset domains would enhance well-being because the self-concordance model suggests that self-consistency positively affects well-being (Sheldon and Elliot, 1999). Gray et al. (2017) meta-analysis suggests that adolescents are much more likely than any other age demographic to experience goal conflict, which highlights the importance of assessing these effects during a period when the self-system is developing.

To date, there is very little research regarding the overlap between mindset domains within people. Meta-analytic findings do suggest small to moderate moderation effects for mindset domain on the associations between mindset and well-being, but these analyses compare domains across studies (Burnette et al., 2020). Moreover, most mindset analyses are typically conducted on a linear level, which may mask non-linear effects (e.g., Dawson, 2014). As such, one line of empirical inquiry in the present study is determining whether congruence is of import to different mindset domains and whether such effects might be interactive or non-linear.

Based on these studies, congruence of mindsets across domains would theoretically lead to positive outcomes, and the relevance of domain-specific mindsets to an individual's identity may influence the impact that any discrepancies or congruencies in mindsets have on the self (Sheldon and Elliot, 1999). Additionally, the literature on mindsets would suggest that congruencies among growth mindsets would be the most beneficial for well-being as growth mindsets are associated with flourishing (Howell et al., 2016; Zhao et al., 2021).

Whereas, most studies to date have examined conflict and unity regarding the *content* of the developing self-system (e.g., conflict between morality and ability goals within the self-system), we propose to examine the effects of incongruencies and congruencies in mindset *processes* across domains (e.g., diverging mindset for morality and ability). Such consideration will inform how harmony and conflict in the processes of the self-system affect well-being. An analytic approach that allows for such investigation is response surface analysis (RSA). Much of the personality literature to date using within-person RSA analyzes congruence and incongruence between desired and actual states in relation to well-being outcomes (e.g., Brandstätter et al., 2016; Verhagen et al., 2018). Such studies typically find that incongruence between desired and actual states is associated with worse well-being, whereas congruence is associated with higher well-being (Verhagen et al., 2018). This type of dissonance between desired and actual states may carry over to dissonance in mindset domains as well. Moreover, RSA allows for the assessment of non-linear effects, which is useful for understanding mindset. It is possible that growth mindsets are not incrementally beneficial. Instead, it might be possible that there are diminishing returns on growth mindset after reaching a certain level, or mid-levels of mindsets might serve as resources because they provide flexibility or as vulnerabilities because they are less coherent. Although exploratory, inspection for such non-linear effects in addition to interactions is overdue in the mindset literature.

### Outcomes of Interest

We selected several domains of outcomes with our mindsets: self-system, personality, self-regulation, and well-being. These domains were pertinent to our interests given the rich literature regarding their associations with individual, specific mindset domains.

#### Self-Systems and Trait Personality

Regarding the self-system, self-efficacy, contingencies of self-worth, and trait personality were assessed. Much of the existing literature points to positive associations between higher growth mindsets and adaptive self-system organization like increased self-efficacy (Komarraju and Nadler, 2013; Diseth et al., 2014). Although mindset is reliably related to self-efficacy, Niiya et al. (2010) found that academic contingent self-worth suppressed the influence of mindsets, such that adolescents with scores across the spectrum of fixed and growth intelligence mindsets engaged in self-handicapping behaviors if their self-worth was contingent on academics. This suggests that contingency of self-worth might suppress or interact with mindset, as both deal with specific domains (Crocker et al., 2003). The present study assessed trait-level personality to compare with mindset, given that we expect mindset to fall into the characteristic adaptation level of personality.

#### Self-Regulation

Mindset is linked with the ability to self-regulate, such that those with higher growth mindsets engage self-regulation more effectively (Burnette and Pollack, 2013). Higher growth mindsets of willpower buffered adverse effects of ego-depletion and emotional dissonance (Konze et al., 2019) and increased self-control in resisting temptations and controlling emotions (Bernecker and Job, 2017). Self-regulation is a central component in people's ability to pursue goals (Sheldon and Kasser, 1998). Because goal consistency leads to better outcomes (Sheldon and Elliot, 1999), it seems likely that congruence across multiple mindset domains may have a positive effect on self-regulatory behaviors and strategies. Emmons et al. (1993) found that goal conflict across domains typically undermines self-regulation, though this has not been explored regarding mindset domains. Thus, this study investigates how congruence or incongruence across ability and moral mindsets affects self-regulatory processes including general regulatory behaviors and self-control.

#### Well-Being

The literature on mindset strongly establishes a link between growth mindset and well-being outcomes like subjective well-being (Zhao et al., 2021), psychological well-being (Zeng et al., 2016), and hedonic and eudaimonic well-being (Howell et al., 2016). In much the same way, more fixed mindsets are associated with worse well-being such as greater negative affect (King, 2016), anxiety, and depression (Schroder et al., 2019). Fear of failure is also associated with higher fixed mindset (Lewis et al., 2020) and can undermine self-image and sense of achievement (Sagar et al., 2007). Growth mindset buffers against negative well-being and increase positive outcomes. In a single-session growth mindset intervention with adolescents, researchers found that adolescents in the intervention group had significant improvements in both self- and parent-reported depression and anxiety compared to the control group (Schleider and Weisz, 2018). As such, anxiety, depression, and fear of failure were assessed in relation to mindsets.

### Current Study

We seek to contribute to the broader discussion of domain specificity and domain generality of mindset while exploring how cohesion and divergence across mindset affect self-system, regulatory, and well-being outcomes. Given that mindset theoretically falls into the characteristic adaptation level of personality, we expect differences across specific domains of mindset. Such exploration will contribute to the literature on personality systems.

We chose to investigate these domains in adolescent participants because such participants are most likely still developing their self-system, which consequently shapes self-regulatory strategies and well-being outcomes. The development of the self-system is characterized by differentiation and hierarchical integration during adolescence (Werner, 1957). Increasing cognitive capacities result in adolescents possessing the ability to differentiate themselves across multiple contexts and domains. At the same time, such differentiation across contexts is pieced into a coherent whole by way of hierarchical integration. Indeed, adolescents with less coherence across domains typically experience self-regulatory deficits, as evinced in behaviors like greater impulsivity (Goth et al., 2012) and tendencies to engage in risky behaviors (Schwartz et al., 2015). In contrast, those with greater coherence and integration usually have a stronger sense of purpose and experience greater subjective well-being (Sumner et al., 2015). Additionally, most mindset intervention studies are directed toward adolescents because this may be a critical period to develop growth mindset (Dweck and Yeager, 2019).

#### Hypotheses

Based on the literature showing that mindsets are associated with goal pursuit processes (including goal setting, pursuit, operating, and monitoring) coupled with the robust literature showing that goal conflict is detrimental for the self and well-being (e.g., Sheldon and Elliot, 1999; Gray et al., 2017), we hypothesize that (*H1*) congruence among the mindset domains will lead to better well-being outcomes. Additionally, based on our conceptualization of mindsets as characteristic adaptations, we hypothesize that (*H2*) mindset domains will demonstrate discrepancies for a majority of participants, indicating domain specificity in line with the characteristic adaptation level of personality, as opposed to dispositional traits. Based on the literature on fixed and growth mindsets, we hypothesize that (*H3*) congruence among the mindset domains with lower fixed mindsets (and thus higher growth mindsets) will be associated with better well-being outcomes.

#### Analytic Plan

**Figure 1:**
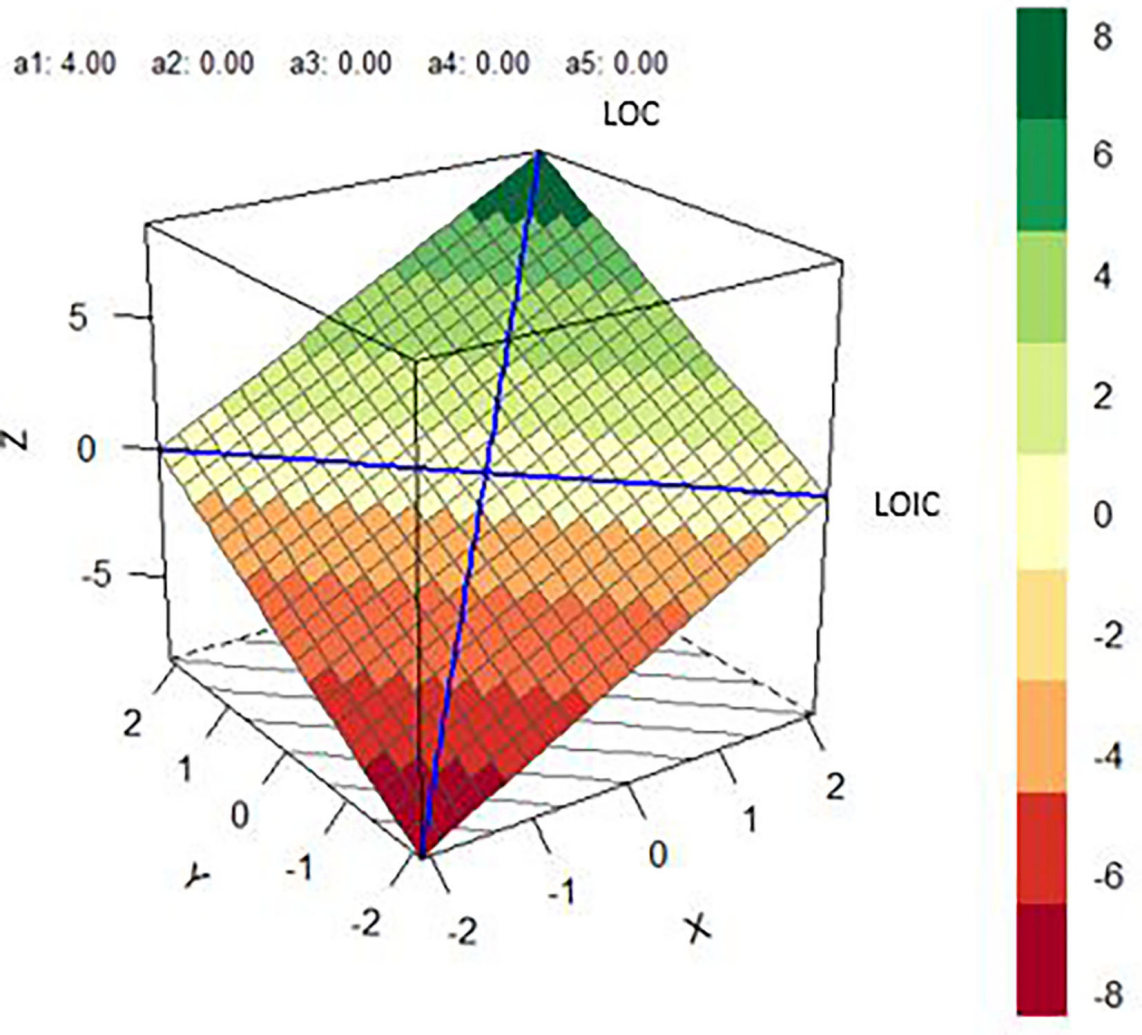
Example response surface analysis.

To assess congruence and discrepancies, we used response surface analysis (RSA). RSA assesses the congruence and incongruence of two variables (X and Y) in relation to a given outcome (Z) using polynomial regression. This places data into a three-dimensional space as X and Y are used to predict Z (see Figure 1 for example RSA plot). This approach is of particular use in the present study, as it allows us to consider the within-person effects of domain specificity across various outcomes. Within the present study, X is moral mindset, and Y is ability mindset; both are assessed in relation to Z, which is represented by different self-system, personality, regulatory, and well-being outcomes in each analysis.

To assess *H1* and *H3*, RSAs were estimated for each pair of moral and ability fixed mindsets in *RStudio* (*R* version 3.6.1) with the RSA Package (Schönbrodt and Humberg, 2020)^1^. This package allowed us to consider how mindset congruence and incongruence were associated with personality traits, self-system indicators, self-regulatory behaviors, and well-being outcomes. RSA produces polynomial regression coefficients (*b*_1_–*b*_5_*)*, which are used to compute the surface coefficients (*a*_1_–*a*_4_*)*, which are then used to compute the effects of (in)congruence on the outcome. The slope and curvature of the line of congruence (LOC) and line of incongruence (LOIC) are reported. The LOC is analyzed with two tests: *a*_1_ and *a*_2_. *A*_1_ (*b*_1_ + *b*_2_) assesses the slope of the LOC with whether an outcome is higher when moral and ability fixed mindset match at higher levels or lower levels. *A*_2_ (*b*_3_ + *b*_4_ + *b*_5_) assesses the curvilinear effect of the LOC with whether an outcome is higher when moral and ability fixed mindset converge at extreme levels or midrange levels. The LOIC is considered with two tests: *a*_3_ and *a*_4_. The slope of LOIC is tested with *a*_3_ (*b*_3_–*b*_4_), which examines whether the outcome is higher when moral fixed mindset is higher than ability fixed mindset or vice versa. *A*_4_ (*b*_3_–*b*_4_ + *b*_5_) tests the curvilinear effect of the LOIC with whether congruence is better or worse than discrepancies in moral and ability fixed mindset. Additionally, following suggestions from Humberg et al. (2019), to determine an overall congruency effect, *p*_10_ must be non-significant, the confidence interval of *p*_11_ must include 1, *a*_4_ must be significantly negative, and *a*_1_*, a*_2_, and *a*_3_ must be non-significant. Specific coefficients of the LOC or LOIC should only be interpreted if there is an overall congruency effect.

In addition to RSAs, to address *H2*, we conducted correlational and confirmatory factor analyses on the two mindset domains to determine the overlap and uniqueness of moral and ability mindset domains.

## Materials and Methods

### Participants

Data were taken from Wave 1 of a 4-wave longitudinal study^2^. Participants (*N* = 618) were recruited via athletic and extracurricular programs at partnering schools in metropolitan southern California. The majority of the sample was female (58.3%). Most participants were older adolescents (*M*_*age*_ = 16.07, *SD*_*age*_ = 0.99) ranging from 15 to 19 years old (35% were 15 years old, 30.7% were 16 years old, 26% were 17 years old, 7.1% were 18 years old, and 0.7% were 19 years old). The sample was ethnically diverse: 42.2% identified as Asian/Asian-American, 29.3% Hispanic, 12.7% Caucasian, 4.8% African American, 0.2% Native American, and 2.4% other. Self-reported socioeconomic status also differed: 10.4% identified as “very poor or poor,” 32.2% “lower middle-class,” 43.8% “middle-class,” and 13.5% “upper middle-class or rich.” Of the participants, 72.5% identified as recently engaging in a sport or competitive activity whereas 27.5% had not. Further demographic information collected as part of the study is available in the Supplementary Table 1.

### Procedure

After receiving approval from the Institutional Review Board, we obtained informed consent from each participant. If the participant was below 18 years of age, we obtained informed consent and assent from their parent or guardian as well, taking into consideration different languages that were spoken by participants' families. Consent and assent forms were available in English, Spanish, Mandarin, and Vietnamese, the primary languages spoken within our sample's population. Participants were emailed a link to the survey via Qualtrics, an online survey platform. The survey took about 50–75 min to complete. Upon completion, participants were thanked and compensated with $14.00 for their time. We report how we determined our sample size, all data exclusions, all manipulations, and all measures in the study in the Supplementary Material. The data that support the findings of this study are openly available in OSF at https://osf.io/3pyuh/.

### Measures

All measures have been previously validated in adolescent and young adult samples.

#### Mindset

Morality and ability mindsets were measured using the six-item Implicit Theories of Morality and Intelligence scale (Dweck et al., 1995) which included two subscales: morality (e.g., “A person's moral character is something very basic about them and it can't be changed much.”) and ability (e.g., “You can learn new things, but you can't really change your basic talent.”). The ability subscale was adapted from the original intelligence subscale; researchers substituted the term “intelligence” with “talent” following Dweck (1996) suggestion to adapt items to specific context. Item responses ranged from 1 (*strongly disagree*) to 6 (*strongly agree*). Most research anchors these items in the opposite direction with 1 representing *strongly agree* and 6 representing *strongly disagree*. However, we chose to do the inverse to keep scale anchoring consistent across all scales administered in the study (i.e., higher scores representing greater agreement with item) because previous surveys with similar participants in the area suggested our adolescent did not always notice the change in anchors. Higher scores indicated greater fixed mindset.

#### Self-System and Personality

##### Personality

Personality was assessed with the Ten-Item Personality Inventory (TIPI; Gosling et al., 2003), which consisted of five subscales: extraversion (e.g., “extraverted, enthusiastic”), agreeableness (e.g., “sympathetic, warm”), conscientiousness (e.g., “dependable, self-disciplined”), emotional stability (e.g., “calm, emotionally stable”), and openness to experiences (e.g., “open to new experiences, complex”). Item responses ranged from 1 (*disagree strongly*) to 7 (*agree strongly*).

##### Contingencies of Self-Worth

Self-systems contingencies were assessed with 10 items of the Contingencies of Self-Worth Scale (Crocker et al., 2003) which included 2 subscales: competition (e.g., “I feel worthwhile when I perform better than others on a task or skill.”) and virtue (e.g., “Doing something I know is wrong makes me lose my self-respect.”). Responses ranged from 1 (*strongly disagree*) to 7 (*strongly agree*). Higher scores on each subscale suggested higher levels of contingent self-worth.

##### Self-Efficacy

Self-efficacy was assessed using 10 items from the General Self-Efficacy Scale (Schwarzer and Jerusalem, 1995), which included items like, “It is easy for me to stick to my aims and accomplish my goals.” Responses ranged from 1 (*not at all true*) to 4 (*exactly true*), with higher scores indicating higher self-efficacy.

#### Self-Regulation

##### Self-Control

Participants were administered the Brief Self-Control Scale (Tangney et al., 2004), a 13-item measure consisting of items such as “I have a hard time breaking bad habits.” Responses ranged from 1 (*not at all*) to 5 (*very much*), so that higher scores indicated lower levels of self-control.

##### Regulatory Behaviors

Regulatory behaviors were measured using the General Regulatory Behavior Questionnaire (Oaten and Cheng, 2006), a 16-item scale that included items like, “how often did you … use social media when you were not supposed to.” Item responses ranged from 1 (*not at all*) to 5 (*almost always*), so higher scores indicated higher dysregulation of behaviors.

#### Well-Being

##### Anxiety

Anxiety was assessed with the seven-item Generalized Anxiety Disorder scale (Spitzer et al., 2006), which consisted of items asking people the extent to which they felt certain symptoms, such as “Feeling nervous, anxious, or on edge.” Item responses ranged from 0 (*not at all*) to 3 (*nearly every day*), with higher scores indicating higher feelings of general anxiety.

##### Fear of Failure

Fear of failure was assessed with the five-item short form of the Performance Failure Appraisal Inventory (Conroy, 2001), which included items like “When I am not succeeding, people are less interested in me.” Item responses ranged from −2 (*do not believe at all*) to +2 (*believe 100% of the time*).

##### Depression

Depression was assessed with 10 items from the Center for Epidemiologic Studies Short Depression Scale (Björgvinsson et al., 2013), which consisted of items like, “I felt depressed.” Response options ranged from 0 (*rarely or none of the time*) to 3 (*most or all of the time*).

## Results

Correlations and measure reliabilities are displayed in Table 1.

**Table 1:** Omegas, means, standard deviations, and correlations among study variables.

**Measure**	**1**.	**2**.	**3**.	**4**.	**5**.	**6**.	**7**.	**8**.	**9**.	**10**.	**11**.	**12**.	**13**.	**14**.	**15**.
1. Moral fixed mindset															
2. Ability fixed mindset	0.35^***^														
3. Extraversion	0.12^**^	0.08													
4. Agreeableness	0.11^*^	0.15^***^	0.00												
5. Conscientiousness	0.04	−0.00	0.17^***^	0.15^***^											
6. Emotional stability	0.05	0.07	0.17^***^	0.19^***^	0.29^***^										
7. Openness to experiences	0.11^*^	0.06	0.31^***^	0.14^**^	0.27^***^	0.10^*^									
8. CSW competition	0.01	0.02	0.00	−0.19^***^	−0.08	−0.05	−0.02								
9. CSW virtue	0.04	0.05	0.09^*^	0.10^*^	0.15^***^	0.05	0.13^**^	0.54^***^							
10. Self–efficacy	0.12^**^	0.04	0.26^***^	0.10^*^	0.48^***^	0.39^***^	0.39^***^	0.09^*^	0.25^***^						
11. Self–control	−0.06	−0.02	−0.10^*^	−0.20^***^	−0.60^***^	−0.36^***^	−0.16^***^	0.16^***^	−0.11^*^	−0.43^***^					
12. Regulatory behavior	−0.10^*^	−0.05	−0.05	−0.19^***^	−0.46^***^	−0.33^***^	−0.14^***^	0.15^***^	−0.08	−0.28^***^	0.65^***^				
13. Anxiety	−0.10^*^	−0.11^*^	−0.12^**^	−0.12^**^	−0.28^***^	−0.57^***^	0.00	0.09	0.03	−0.29^***^	0.38^***^	0.34^***^			
14. Fear of failure	−0.10^*^	−0.08	−0.12^**^	−0.06	−0.17^***^	−0.30^***^	−0.10^*^	0.33^***^	0.25^***^	−0.24^***^	0.28^***^	0.26^***^	0.26^***^		
15. Depression	−0.04	−0.10^*^	−0.23^***^	−0.10^*^	−0.31^***^	−0.54^***^	−0.11^**^	0.08	−0.07	−0.30^***^	0.39^***^	0.39^***^	0.38^***^	0.37^***^	
Mean	3.70	3.88	4.03	4.54	4.66	4.34	5.07	5.07	4.81	2.98	3.03	2.56	1.99	3.40	1.88
Standard deviation	1.06	1.35	1.43	0.99	1.28	1.37	1.17	1.08	0.96	0.44	0.67	0.51	0.67	0.91	0.54
Omega reliability	0.81	0.87	–	–	–	–	–	0.81	0.96	0.85	0.83	0.74	0.84	0.80	0.81

*Extraversion, Agreeableness, Emotional Stability, and Openness are measured using two-item scales, and therefore omega reliability could not be calculated. CSW = contingencies of self-worth*.

* *p < 0.05*,

** *p < 0.01*,

*** *p < 0.001*.

### Power

For response surface analysis (RSA), sample size is determined by having at least 2–3 times as many participants as would be needed to detect linear main effects (Aiken and West, 1991; Humberg et al., 2019). A priori calculations from G^*^Power indicated that to test linear multiple regression with two predictors and a single outcome with a small effect (*f*^2^ = 0.02), a total sample of 311 participants was required. We chose a small effect as there are no extant studies or established effect sizes for the analyses being completed in the present research. Upon doubling this linear main effect for RSA, a total number of 622 participants was required. We were a few participants shy of this number.

### Mindset Discrepancies and Functioning

Moral and ability fixed mindsets were congruent for 34.6% (|Δ*z* < 0.5|) of the adolescent participants; moral fixed mindsets were higher than ability fixed mindsets for 28.8% of participants; and ability fixed mindsets were higher than moral fixed mindsets for 36.6% of participants.

### Mindset Confirmatory Factor Analyses

**Figure 2:**
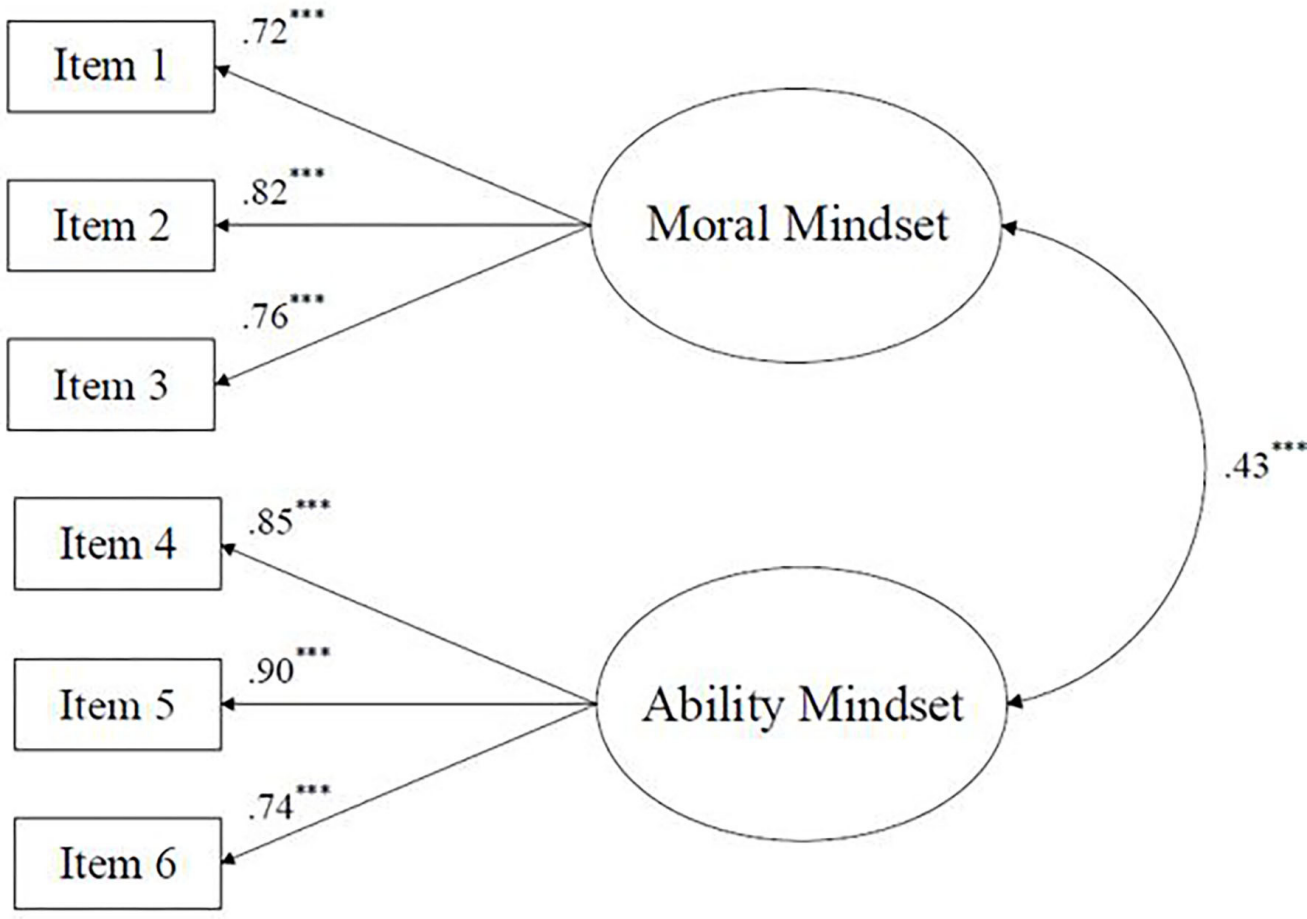
Confirmatory factor analysis of mindset. *N* = 599. Standardized values used. Model fit, χ^2^ (8) = 24.89, *p* < 0.01, CFI = 0.99, TLI = 0.98, RMSEA = 0.06, and SRMR = 0.03. ***p < 0.001.

To further support that these mindset domains are distinct but associated constructs, we ran confirmatory factor analyses (CFA) in *Mplus 8.4* (Muthén and Muthén, 1998–2017) to examine the structure of mindset in our sample. Model fit for mindset was good, χ^2^ (8) = 24.89, *p* < 0.01, CFI = 0.99, TLI = 0.98, RMSEA = 0.06, and SRMR = 0.03. All items loaded highly and significantly onto their respective latent factors and can be viewed in Figure 2. Moral and ability mindsets were moderately and significantly associated (standardized *r* = 0.43), lending support to these domains being related but distinct constructs.

### Congruency Effects Analysis

To determine the role of moral and ability mindsets in predicting the outcomes of interest (Z), hierarchical regressions were conducted before full response surface analyses. Doing so identified if there are any additive or multiplicative effects prior to congruency analyses. As can be viewed in the first two rows for each outcome variable in Table 2 there were only a few significant effects of moral and ability mindset. For extraversion, openness to experiences, and self-efficacy, moral fixed mindset alone was a positive additive predictor such that when moral fixed mindset increased, so too did extraversion, openness to experiences, and self-efficacy. Ability fixed mindset did not predict any outcomes in the additive model. There were no significant effects for moral mindset multiplied with ability mindset or for ability mindset squared. Moral fixed mindset squared negatively predicted competition self-worth, such that when moral fixed mindset squared increased, competition self-worth decreased.

**Table 2:** Moral vs. ability mindsets across outcome variables.

	**Estimated regression model**						**Position of 1st Principal Axis (PA)**			**LOC**		**LOIC**	
**Outcome**	***b_**0**_***	***b_**1**_***	***b_**2**_***	***b_**3**_***	***b_**4**_***	***b_**5**_***	***p_**10**_***	***p_**11**_***	***R^**2**^***	***a1***	***a2***	***a3***	***a4***
	**Intercept**	**Moral**	**Ability**	**Moral^**2**^**	Moral*Ability	**Ability^**2**^**	**Intercept**	**Slope**		**Slope**	**Curvilinear Effect**	**Slope**	**Curvilinear Effect**
								**of 1 PA**		**of LOC**	**of LOC**	**of LOC**	**of LOIC**
Extraversion	3.34^***^	0.10^*^	0.05						0.02^*^				
	3.28^***^	0.09	0.10	−0.00	0.01	−0.05			0.02				
	4.00^***^	0.13^*^	0.06	0.00	0.00	−0.01	4.23	−0.01	0.02	0.19^**^	−0.01	0.07	−0.01
Agreeableness	3.96^***^	0.06	0.13						0.03^***^				
	3.95^***^	0.10	0.10	0.02	−0.05	0.03			0.03^*^				
	4.50^***^	0.06	0.10^*^	−0.01	0.00	0.00	−29.14	7.63	0.03	0.15^**^	−0.00	−0.04	−0.01
Conscientiousness	4.51^***^	0.05	−0.02						0.00				
	5.19^***^	−0.26	−0.01	0.43	0.07	−0.29			0.01				
	4.67^***^	0.03	−0.03	0.01	0.07	−0.04	−0.16	0.55	0.01	0.03	0.05	0.03	−0.10
Emotional Stability	3.95^***^	0.03	0.06						0.01				
	4.42^***^	−0.43	0.37	−0.04	0.48	−0.28			0.01				
	4.28^***^	0.01	0.09	0.08	−0.01	−0.04	1.21	−0.03	0.01	0.10	0.04	−0.08	0.06
Openness to Experiences	4.58^***^	0.10^*^	0.03						0.01^*^				
	5.27^***^	−0.03	−0.30	−0.01	0.14	0.34			0.02				
	4.95^***^	0.11	0.01	0.02	−0.00	0.04	−41.81	−16.36	0.02	0.11	0.06	0.10	0.06
Competition CSW	5.01^***^	0.01	0.01						0.00				
	5.06^***^	−0.02	−0.07	−0.62^**^	0.38	0.49			0.02				
	4.96^***^	0.03	−0.01	0.05	−0.09^*^	0.05	−1.31	−1.00	0.02	0.02	0.01	0.04	0.19^**^
Virtue CSW	4.60^***^	0.03	0.05						0.00				
	4.69^***^	0.14	−0.22	−0.18	−0.02	0.39			0.01				
	4.75^***^	0.04	0.01	−0.00	−0.02	0.04	14.12	−3.48	0.01	0.05	0.01	0.03	0.06
Self–Efficacy	2.79^***^	0.12^*^	0.00						0.01^*^				
	3.10^***^	−0.06	−0.33	0.07	0.15	0.29			0.02				
	2.93^***^	0.05^*^	−0.01	0.01	0.00	0.01	7.90	2.34	0.02	0.04	0.03	0.05	0.02
Self–Control	3.18^***^	−0.06	−0.00						0.00				
	2.57^***^	0.22	0.35	−0.40	−0.06	−0.10			0.02				
	3.07^***^	−0.03	0.01	−0.01	−0.04	−0.01	−0.46	−0.97	0.02	−0.02	−0.05^*^	−0.03	0.02
Regulatory Behavior	2.75^***^	−0.09	−0.02						0.01				
	2.26^***^	0.23	0.31	−0.41	−0.11	−0.07			0.02^*^				
	2.60^***^	−0.04	−0.00	−0.01	−0.03	−0.00	−1.11	−1.13	0.02	−0.04	−0.04^*^	−0.03	0.02
Anxiety	2.32^***^	−0.08	−0.08						0.02^*^				
	2.35^***^	−0.17	−0.01	−0.15	0.18	0.03			0.02				
	2.00^***^	−0.05	−0.04	0.02	−0.01	0.00	−30.01	−0.40	0.02	−0.09^**^	0.00	−0.01	0.03
Fear of Failure	3.79^***^	−0.08	−0.05						0.01^*^				
	3.59^***^	−0.21	0.29	−0.07	0.16	−0.30			0.02				
	3.45^***^	−0.08	−0.02	0.02	−0.01	−0.03	−0.47	−0.09	0.02	−0.10^*^	−0.02	−0.06	0.00
Depression	2.05^***^	−0.01	−0.09						0.01				
	1.88^***^	0.20	−0.14	−0.33	−0.02	0.27			0.01				
	1.89^***^	0.01	−0.04	−0.00	−0.02	0.01	−2.17	−1.83	0.01	−0.04	−0.01	0.05	0.04

*CSW, Contingencies of Self-Worth*.

* *p < 0.05*

** *p < 0.01*

*** * p < 0.001*.

Although it is common to stop RSA analyses when *b*_3_*-b*_5_ are non-significant, we still calculated full RSA parameters for all variables, which are displayed in the third row for each outcome variable. None of the RSAs for the outcomes met the requirements to evince a congruence effect. However, coefficients *a*_1_*, a*_2_, and *a*_4_ suggest some potential lines of inquiry for investigators, so we report them. Although interpreted in congruence language, it is important for readers to recognize that there were not overall effects for congruence supported. For both extraversion and agreeableness, the significance of *a*_1_ indicated an effect on the slope of the LOC, suggesting that extraversion and agreeableness were higher when moral and ability fixed mindsets matched at higher levels. For competition contingent self-worth, the significance of *a*_4_ suggested a curvilinear effect of the LOIC. That is, competition contingent self-worth was higher when moral and ability fixed mindsets matched than when they mismatched. For self-control and general regulatory behaviors, the significance of indicated a curvilinear effect for LOC. That is, both self-dysregulation and general dysregulation were higher when moral and ability fixed mindsets were more congruent at midrange levels than when they matched at extreme levels. For both anxiety and fear of failure, the significance of *a*_1_ indicated an effect on the slope of the LOC. Both anxiety and fear of failure were higher when moral and ability fixed mindsets matched at lower levels. However, for all these effects, it must be reiterated that an overall congruency effect was not supported.

## Discussion

The present study contributes to the larger literature in several compelling ways. Interestingly, *H1* was not supported; there were no overall congruency effects for any of the models (Humberg et al., 2019). This indicates that overall congruency between moral and ability mindsets did not correlate with better or worse outcomes. Additionally, the data suggest mindsets are characteristic adaptations because of their high levels of domain specificity; confirmatory factor analysis supported distinctions between moral and ability mindset, and two-thirds of the participants demonstrated discrepancies in mindset domains, which supports *H2*. Although we could not fully examine *H3* because of a lack of congruency effects, examinations of coefficients related to the line of congruence suggest important potential avenues of future research.

In our sample relations among mindset domains and outcomes of interest were lower than expected or found previously. For example, most literature finds a positive association between depression and fixed mindset (e.g., Schleider and Weisz, 2018); however, in our study depression was negatively associated with ability fixed mindset (*r* = −0.10) and not significantly associated with moral fixed mindset at all. Given that linear effects can mask non-linear effects, we still ran the analyses proposed in our hypotheses (Dawson, 2014).

### Hypothesis 1: Congruency Between Mindset Domains Bears No Effect on Well-Being Outcomes

Contrary to *H1*, congruency between moral and ability mindsets did not significantly affect any of the well-being outcomes. The null results for congruence contrast with other literature related to characteristic adaptation constructs suggesting that (in)congruence between goals matters (Sheldon and Elliot, 1999; Gray et al., 2017). Indeed, much of extant literature on goals suggest that conflict between domains inhibits well-being and congruence enhances well-being. This finding was not reflected in the present study. Instead, it is most likely that ability and moral mindsets are distinct qualities for which congruence is not of import for well-being.

Beyond the simple explanation that congruence among domain-specific mindsets is not relevant for well-being, there are several possible explanations for the lack of an overall congruence effect. It may be possible that congruence is not the best way to model effects across mindset domains as it does not resemble congruency found between implicit and explicit expressions of the same constructs such as self-esteem (Lupien et al., 2010) or motives (Thielgen et al., 2015). In such cases, both the explicit and implicit construct reflect an underlying dimension, which does not seem to be the same case for mindset. Additionally, recent literature on other characteristic adaptation constructs (e.g., character strengths) has shifted away from attempting to assess consistency across contexts and instead focused toward assessing coherence, wherein a person demonstrates appropriate character mechanisms in varying amounts and in varying contexts (Lerner, 2019; Nucci, 2019). Perhaps mindset, like character, should be considered from a coherence framing rather than consistency framing.

It is also possible that the adolescents in the study may still be engaged in the differentiation stage of self-system development and may not have moved into the integration stage (Werner, 1957). Werner (1957) orthogenetic principle suggests that as development occurs, there is a process from a state of globality and lack of differentiation to a state of increasing differentiation, articulation, and integration. Returning again to the example of character, researchers have found that global character becomes more differentiated and specific as children become adolescents (Shubert et al., 2019). Mindset may follow a similar trajectory, the domains becoming more differentiated with age before later integration. Domain incongruence could be further explored through comparing the centrality of the various domains to the specific person as well as the hierarchic integration of the domains (Werner, 1957). Much of the recent literature exploring integration focuses on emerging adults rather than adolescents, perhaps, because it is more normative for integration to take place in emerging adulthood (Schwartz et al., 2015; Sumner et al., 2015). Future studies should examine samples spanning from early adolescence into adulthood to examine age differences in congruence effects.

Another potential explanation is that the measures of each mindset domain tap into other constructs besides pure mindset. The items involved in assessing moral mindset (e.g., “A person's moral character is something very basic about them and it can't be changed much”) could tap into the broader construct of moral identity. Moral identity serves to pair moral motivation and reasoning with actual behavior (Hardy and Carlo, 2011). Given that growth mindset has consistently been linked with increased motivation (Yeager and Dweck, 2012; Han et al., 2020), moral mindset may assess components of moral identity. It is apparent that the moral and ability mindsets tap into specific domains.

### Hypothesis 2: Mindsets Within the Personality System Are Largely Domain Specific, Suggesting They Are Characteristic Adaptations

Lending support to *H2*, 65.4% of participants demonstrated discrepancies in ability and moral mindsets, suggesting a high level of domain specificity. It is important to note that there was a modest correlation between ability and moral mindsets (*r* = 0.35 for basic correlation; *r* = 0.43 for CFA); however, despite their association, these domains were distinct from each other for nearly two-thirds of the participants. Returning to McAdams and Pals's (2006) conceptualization of characteristic adaptations, a key aspect of these personality units is that they are “contextualized in time, place, and/or social role” (p. 208). This contextualization is the central separation between characteristic adaptations and dispositional traits, in that dispositional traits are typically broad and decontextualized.

Situating mindsets as characteristic adaptations is further supported by their relations with the Big Five traits in this study. The mindsets were clearly distinct from the Big Five traits, echoing previous literature (Burnette and Pollack, 2013). Both moral and ability fixed mindsets were positively correlated with extraversion, agreeableness, and openness to experiences, but effects were small indicating distinctiveness. Moreover, the lack of congruence effect for personality traits further supports their differentiation at a different level of personality.

### Null Results Related to Hypothesis 3, but Interesting Exploratory Findings

Given that there was no overall congruence effect, we cannot make any actual conclusions about how higher or lower fixed mindsets accompanying congruence relate to well-being to address *H3*. However, significant effects related to the line of congruence suggest interesting points of discussion that may be useful for spurring future research. These findings should be interpreted with extreme caution given their exploratory nature.

#### Exploratory Findings Suggest Congruencies With Lower Fixed Mindset Are Sometimes Associated With Lower Well-Being

Although there were no overall congruence effects, results suggest both anxiety and fear of failure, indicators of well-being, are higher when participants' mindsets are congruent in the direction of a higher growth mindset. This finding means that possessing a growth mindset across multiple mindset domains is associated with *higher* arousal and reactivity. This may be because people with more fixed mindsets are more likely to make external attributions regarding ability and failure (Bodill and Roberts, 2013), which could relieve anxiety and fear of failure. Attributions regarding potential failure would be external and, thus, would not be the responsibility of the participant. In essence, those with fixed mindsets may believe their performance, good or poor, is no longer a result of their own innate ability, which alleviates anxiety. In a similar manner, *H3* was not supported with the personality indicators. Higher levels of extraversion and agreeableness were associated with more fixed mindsets than growth mindsets. Given the unexpected nature of this finding, further exploration and consideration are necessary.

#### Exploratory Findings Suggest Moderate Mindsets May Be Detrimental

Results from the self-dysregulation variable suggest that congruency in mindset at either extreme—either fixed or growth—rather than midrange levels predicted greater capacity to self-regulate. In other words, participants who were congruent at more extreme ends of the poles experienced better outcomes than those who were congruent at mid-range levels for regulation^3^. This finding suggesting a potential cost of a “neutral” mindset may have serious implications for intervention research. Interventions aimed toward shifting a participant from a more fixed mindset to a more growth mindset might leave that participant in a sort of middle ground. In light of the findings in the present study, such a move could potentially be more harmful than staying at a fixed mindset. Our exploratory findings, if confirmed, would suggest future intervention work should carefully ensure that participants will not be left in a potentially worse state than the beginning of the intervention, perhaps by ensuring that the intervention fully moves participants into a growth mindset state. However, these findings are purely exploratory in nature because there was no overall congruence effect, but they merit discussion to spur future inquiry.

### Constraints on Generality

The present study involved a demographically diverse subject pool recruited from high schools in metropolitan southern California, meaning replicability of the findings will likely depend on similarity to the sample. A high proportion of participants were Asian and Latinx (70.6% of sample), which are typically culturally interdependent. Some research suggests that mindsets act universally across cultures (e.g., Church et al., 2003); however, some findings indicate that individualistic groups are more susceptible to fixed mindsets (e.g., Church et al., 2012), and collectivistic groups engage higher growth mindsets (e.g., Tang et al., 2016). There is no research on the cultural or ethnic differences of mindset congruence across multiple domains, which makes it difficult to make any predictions about generalizability of findings in a different sample. Most participants were student athletes (71.9%). Because characteristic adaptations depend largely on the centrality and relevance to one's personal identity (Sheldon and Elliot, 1999), the variables related to performance (e.g., competition contingency self-worth) may not be intrinsically important to people outside competitive contexts. Analyses were run (available in Supplementary Material) on differences in mindset based on race, culture, and athletic status, which were non-significant. The analysis comparing typically collectivistic cultures with individualistic cultures approached significance (*p* = 0.052) for ability mindset, such that participants from typically collectivistic cultures displayed lower fixed mindsets, in line with previous literature (Church et al., 2012; Tang et al., 2016). As previously discussed, there is good reason to expect we might find different results in adult samples. We have no reason to believe that the results depend on other characteristics of the participants, materials, or context.

### Limitations and Future Directions

The present findings should be interpreted in consideration of several limitations. First, the study was slightly underpowered. Future research utilizing response surface analyses on mindset domain congruency should utilize a larger sample size in order to best detect effects that exist in the data. Secondly, the present findings consider the relations among constructs only at a single time point. Future research should consider these associations across time to investigate the bidirectional relations of characteristic adaptations and dispositional traits (McAdams and Pals, 2006; McCrae et al., 2008).

Finally, congruencies and discrepancies between mindsets were limited to two specific domains: morality and ability. Ability mindset could arguably cover a broader range of specific ability-akin domains like intelligence or athletic ability. Moreover, there is little research on moral mindset, especially in relation to our outcomes of interest. Because mindsets exist within and across a vast range of domains, further research is needed to investigate congruency patterns among other specific domains of mindset (e.g., intelligence, personality; Dweck and Yeager, 2019).

## Conclusion

Consistent with Dweck and Yeager (2019) conceptualization of mindset, these results provide empirical support that mindsets are domain specific and should be categorized at the characteristic adaptation level of personality. Contrary to hypotheses based on self-concordance theory (Sheldon and Elliot, 1999) and research on goal conflict (Gray et al., 2017), we did not find that congruence in mindsets across domains affects well-being in our sample. These findings suggests that congruence matters for the content of the developing self-system across domains but not necessarily for mindset processes of the self-system among adolescents. Within the purview of the present study, we failed to reject the null hypothesis. Future research is needed to test whether the null effects for mindset congruence hold in another sample of adolescents. Should the non-significant findings hold true, one could explore whether this is true only for those still navigating differentiation and integration of the self-system (Werner, 1957) or whether it would generalize into adulthood.

## Data Availability Statement

The datasets generated for this study can be found in online repositories. The names of the repository/repositories and accession number(s) can be found below: Open Science Framework https://osf.io/3pyuh/.

## Ethics Statement

The studies involving human participants were reviewed and approved by Fuller Theological Seminary's Institutional Review Board. Written informed consent to participate in this study was provided by the participants' legal guardian/next of kin.

## Author Contributions

JR contributed to conceptualization, data curation, data analyses, and writing the manuscript. EW contributed to writing the manuscript. LB contributed to conceptualization, data analyses, and data curation. BH contributed to conceptualization and funding acquisition. SS contributed to conceptualization, editing the manuscript, funding acquisition, and overseeing the project. All authors contributed to the article and approved the submitted version.

## Conflict of Interest

The authors declare that the research was conducted in the absence of any commercial or financial relationships that could be construed as a potential conflict of interest.

## Footnotes

^1^Analyses were not pre-registered.

^2^Other waves of data were not considered as they were administered after experimental manipulations that could affect mindset.

^3^Indeed, there seems an implicit recognition of the problems of a neutral mindset in mindset measurement, which tends to assess mindset using a 6-point Likert system from 1 (*strongly agree*) to 6 (*strongly disagree*). Several researchers suggest that this 6-point anchoring system is used to avoid a “neutral” mindset; participants will always lean at least slightly toward growth or fixed mindsets (Burnette and Pollack, 2013; Cainëls et al., 2017). This measurement strategy may have some bearing, given the findings with self-dysregulation.
