# Supplementary material for: Mindset as Characteristic Adaptations: Using Response Surface Analysis to Assess Mindset in the Personality System
Source: Front Psychol. 2021 Jul 20;12:701510. doi: 10.3389/fpsyg.2021.701510 (PMC8329028; doi:10.3389/fpsyg.2021.701510)
Supplement: Supplementary file 1 [file Data_Sheet_1.DOCX]

**Online Supplemental Materials**

Mindset as Characteristic Adaptations: Using Response Surface Analysis to Assess Mindset in the Personality System

**Table of Contents**

[**Sample Size and Data Exclusions** 2](#_Toc74212240)

[**Athletes vs. Non-Athletes** 3](#_Toc74212241)

[**Ethnic Differences** 4](#_Toc74212242)

[**Measure Items** 5](#_Toc74212243)

[**Excluded Measures** 9](#_Toc74212244)

[**References** 10](#_Toc74212245)

[**Supplemental Table 1.** *Demographic Breakdown of Participants* 12](#_Toc74212246)

[**Response Surface Analysis *R* Syntax** 13](#_Toc74212247)

# **Sample Size and Data Exclusions**

**Sample Size**

Given the difficulties of recruiting participants in this setting, lack of clear a priori effect sizes, and potential attrition, we recruited as many participants as possible among high schools and teams where we had access.

**Data Exclusions**

No data were excluded from the dataset. Participants who did not have any responses for independent variables in the present study (*n* = 156) were not included in analyses.

**Manipulations**

No manipulations were present at the Time 1 survey, from which all analyses were conducted. Upon completing the first survey, participants were assigned to one of six framing conditions crossing three purpose domains with the approach vs. avoidance orientation (spiritual approach, spiritual avoidance, moral approach, moral avoidance, instrumental approach, instrumental avoidance).

# **Athletes vs. Non-Athletes**

Of the sample, 70.6% of participants claimed to have recently been involved in a sport or competitive activity, compared to 27.6% who had not and 1.8% who did not respond. Since we were analyzing participants within their context for ability mindset, we ran *t*-tests comparing those who recently participated in a sport to those who did not on ability mindset. Participants did not differ on ability mindset based on being engaged in athletic activities or not, *t* = 1.05, *p* = .29. Additionally, participants did not differ on moral mindset, *t* = 0.58, *p* = .56. As such, we felt confident in running the analyses on the entire sample rather than just the athlete subsample.

# **Ethnic Differences**

Given that our sample was majority Asian and Latinx (70.6%), we ran an ANOVA to compare ethnic groups on moral and ability mindsets. Participants did not differ on moral mindset (*F* = 1.34, *p* = .24) or ability mindset (*F* = 1.54, *p* = .16). Because of what we mentioned in our constraints on generality, we also ran a *t*-test to compare culturally collectivistic participants (e.g., Asian, Latinx) with culturally individualistic participants (e.g., Caucasian) on mindsets. Participants did not differ on moral mindset (*t* = 0.35, *p* = .73), however it approached significance on ability mindset (*t* = 1.95, *p* = .052), such that participants from typically collectivistic cultures displayed lower fixed mindsets than those from culturally individualistic cultures, following trends in cross-cultural studies (e.g., Church et al., 2012; Tang et al., 2016).

# **Measure Items**

**Implicit Theories of Morality and Intelligence Scale (Dweck et al., 1995)**

Please indicate your level of agreement with the statements below.

1. A person’s moral character is something very basic about them and it can’t be changed much

2. Whether a person is responsible and sincere or not is deeply ingrained in their personality. It cannot be changed very much.

3. There is not much that can be done to change a person’s moral traits (e.g., conscientiousness, uprightness, and honesty)

4. You have a certain amount of talent and you really can’t do much to change it

5. Your talent is something about you that you can’t change much

6. You can learn new things, but you can’t really change your basic talent

*Moral Fixed Mindset*: 1, 2, 3

*Ability Fixed Mindset:* 4, 5, 6

**Ten-Item Personality Inventory (Gosling et al., 2003)**

Here are a number of personality traits that may or may not apply to you. Please rate each statement. I see myself as…

1. extraverted, enthusiastic

2. critical, quarrelsome

3. dependable, self-disciplined

4. anxious, easily upset

5. open to new experiences, complex

6. reserved, quiet

7. sympathetic, warm

8. disorganized, careless

9. calm, emotionally stable

10. conventional, uncreative

*Reverse-scored:* 2, 4, 6, 8, 10

*Extraversion:* 1, 6

*Agreeableness*: 2, 7

*Conscientiousness*: 3, 8

*Emotional Stability*: 4, 9

*Openness to Experience:* 5, 10

**Contingencies of Self-Worth Scale (Crocker et al., 2003)**

Please respond to each of the following statements by selecting your answer using the scale from “Strongly disagree” to “Strongly agree.” If you haven’t experienced the situation described in a particular statement, please answer how you think you would feel if that situation occurred.

1. I feel worthwhile when I perform better than others on a task or skill

2. Knowing that I am better than others on a task raises my self-esteem

3. Doing something I know is wrong makes me lose my self-respect

4. My self-esteem would suffer if I didn’t have God’s love

5. Doing better than others gives me a sense of self-respect

6. My self-esteem goes up when I feel that God loves me

7. When I think that I’m disobeying God, I feel bad about myself

8. I feel worthwhile when I have God’s love

9. My self-worth is affected by how well I do when I am competing with others

10. Whenever I follow my moral principles, my sense of self-respect gets a boost

11. My self-worth is based on God’s love

12. My self-esteem depends on whether or not I follow my moral/ethical principles

13. I couldn’t respect myself if I didn’t live up to a moral code

14. My self-esteem would suffer if I did something unethical

15. My self- worth is influenced by how well I do on competitive tasks

*Competition Contingent Self-Worth:* 1, 2, 5, 9, 15

*God’s Love Contingent Self-Worth:* 4, 6, 7, 8, 11

*Virtue Contingent Self-Worth*: 3, 10, 12, 13, 14

**General Self-Efficacy Scale (Schwarzer & Jerusalem, 1995)**

Please rate your agreement with the following statements.

1. I can always manage to solve difficult problems if I try hard enough.

2. If someone opposes me I can find the means to get what I want.

3. It is easy for me to stick to my aims and accomplish my goals.

4. I am confident that I could deal efficiently with unexpected events.

5. Thanks to my resourcefulness, I know how to handle unforeseen situations.

6. I can solve most problems if I invest the necessary effort.

7. I can remain calm when facing difficulties because I can rely on my coping abilities.

8. When I am confronted with a problem, I can usually find several solutions.

9. If I am in trouble, I can usually think of a solution.

10. I can usually handle whatever comes my way.

11. I can always manage to solve difficult problems if I try hard enough.

**Brief Self-Control Scale (Tangney et al., 2004)**

Using the scale provided, please indicate how much each of the following statements reflects how you are.

1. I am good at resisting temptation

2. I have a hard time breaking bad habits

3. I am lazy

4. I say inappropriate things

5. I do certain things that are bad for me, if they are fun

6. I refuse things that are bad for me

7. I wish I had more self-discipline

8. People would say that I have iron self-discipline

9. Pleasure and fun sometimes keep me from getting work done

10. I have trouble concentrating

11. I am able to work effectively towards long-term goals

12. Sometimes I can’t stop myself from doing something, even if I know it is wrong

13. I often act without thinking through all the alternatives

*Reverse-scored:* 1, 6, 8, 11

**General Regulatory Behavior Questionnaire (Oaten & Cheng, 2006)**

In the last three days how often did you…

1. use social media when you were not supposed to

2. eat junk food

3. clean up after yourself

4. practice healthy habits

5. turn off lights when you left a room

6. spend money impulsively

7. turn assignments in on time

8. over-spend

9. lose your temper

10. skip studying to hang out with friends

11. arrive to class late

12. watch TV instead of studying

13. put something off

14. leave dishes in the sink

15. floss your teeth

16. sleep in

*Reverse-scored:* 3, 4, 5, 7, 15

**Generalized Anxiety Disorder Scale (Spitzer et al., 2006)**

Over the last two weeks, how often have you been bothered by the following problems?

1. Feeling nervous, anxious, or on edge.

2. Not being able to stop or control worrying

3. Worrying too much about different things

4. Trouble relaxing

5. Being so restless that it’s hard to sit still

6. Becoming easily annoyed or irritable

7. Feeling afraid as if something awful might happen

**Performance Failure Appraisal Inventory (Conroy, 2001)**

Please rate how often you believe the following statements.

1. When I am failing, I am afraid that I might not have enough talent

2. When I am failing, it upsets my “plan” for the future

3. When I am not succeeding, people are less interested in me

4. When I am failing, important others are disappointed

5. When I am failing, I worry about what others think about me

**Center for Epidemiologic Studies Short Depression Scale (Björgvinsson et al., 2013)**

Using the scale below, indicate the number which best describes how often you felt or behaved this way.

1. I felt depressed.

2 .I felt that everything I did was an effort.

3. My sleep was restless.

4. I was happy.

5. I felt lonely.

6. People were unfriendly.

7. I enjoyed life.

8. I felt sad.

9. I felt that people disliked me.

10. I could not get “going”

*Reverse-scored*: 4, 7

# **Excluded Measures**

The following measures were excluded from analysis: 3-Factor Patience Questionnaire (Schnitker, 2012); Children’s Sadness and Anger Management Scale (Zeman et al., 2001); Duke University Religion Index (Koenig & Bussing, 2010); Satisfaction with Life Scale (Diener et al., 1985); Positive and Negative Affect Schedule (Ebesutani et al., 2012); UCLA Loneliness Scale (Hays & DiMatteo, 1987); Youth Risk Behavior Surveillance (Foti et al., 2011); Meaning in Life Questionnaire (Strenger et al., 2006); and Group Entitativity Measure (Gaertner & Schopler, 1998).

Several of these measures (e.g., Group Entitativity Measure) were excluded as they did not fit our research aims in this paper. Several measures (e.g., Meaning in Life Scale; Satisfaction with Life Scale) were excluded because they lacked association with either mindset domain.

# **References**

Aiken, L. A., & West, S. G. (1991). *Multiple regression: Testing and interpreting interactions.* Thousand Oaks, CA: Sage.

Björgvinsson, T., Kertz, S. J., Bigda-Peyton, J. S., McCoy, K. L., & Aderka, I. M. (2013). Psychometric properties of the CES-D-10 in a psychiatric sample. *Assessment*, *20*(4), 429-436. <https://doi.org/10.1177/1073191113481998>

Conroy, D. E. (2001). Progress in the development of a multidimensional measure of fear of failure: The Performance Failure Appraisal Inventory (PFAI). *Anxiety, Stress and Coping*, *14*(4), 431-452.

Crocker, J., Luhtanen, R., Cooper, M., Bouvrette, A., & Crocker, J. (2003). Contingencies of self-worth in college students: Theory and measurement. *Journal of Personality and Social Psychology*, *85*(5), 894–908.

Gosling, S. D., Rentfrow, P. J., & Swann, W. B. (2003). A very brief measure of the Big-Five personality domains. *Journal of Research in Personality, 37*(6), 504-528.

Mrazek, A. J., Ihm, E. D., Molden, D. C., Mrazek, M. D., Zedelius, C. M., & Schooler, J. W. (2018). Expanding minds: Growth mindsets of self-regulation and the influences on effort and perseverance. *Journal of Experimental Social Psychology*, *79*, 164-180.

Oaten, M., & Cheng, K. (2006). Improved self-control: The benefits of a regular program of academic study. *Basic and Applied Social Psychology*, *28*(1), 1-16.

Schönbrodt, F. D., & Humberg, S. (2020). RSA: An *R* package for response surface analysis (version 0.10.1). https://cran.r-project.org/package=RSA

Spitzer, R. L., Kroenke, K., Williams, J. B.W., & Löwe, B. (2006). A brief measure for assessing generalized anxiety disorder: The GAD-7. *Archive of Internal Medicine*, *166*(10), 1092–1097.

Tangney, J. P., Baumeister, R. F. and Boone, A. L. (2004). High self‐control predicts good adjustment, less pathology, better grades, and interpersonal success. *Journal of Personality*, *72*, 271-324.

# **Supplemental Table 1.** *Demographic Breakdown of Participants*

| Demographics | Percent | Mean | SD |
| --- | --- | --- | --- |
| Age |  | 16.07 | 0.99 |
| Gender |  |  |  |
| Female | 56.6% |  |  |
| Male | 42.0% |  |  |
| Transgender | 0.4% |  |  |
| Ethnicity |  |  |  |
| Asian/Asian American | 42.2% |  |  |
| African/African American | 4.8% |  |  |
| Latino/a | 29.3% |  |  |
| Native American/First Nation | 0.2% |  |  |
| White/Caucasian | 12.7% |  |  |
| Two or More | 8.4% |  |  |
| Other | 2.4% |  |  |
| Socioeconomic Status |  |  |  |
| Very Poor | 0.8% |  |  |
| Poor | 9.7% |  |  |
| Lower Middle-Class | 32.5% |  |  |
| Middle-Class | 43.5% |  |  |
| Upper Middle-Class | 13.1% |  |  |
| Upper-Class/Rich | 0.4% |  |  |
| Grade |  |  |  |
| High School Freshman | 20.6% |  |  |
| High School Sophomore | 24.1% |  |  |
| High School Junior | 32.2% |  |  |
| High School Senior | 22.0% |  |  |
| Other | 1.0% |  |  |
| Religious Tradition |  |  |  |
| Atheism | 5.8% |  |  |
| Buddhism | 12.9% |  |  |
| Christianity/Catholic | 41.8% |  |  |
| Christian/Latter-Day Saints | 0.2% |  |  |
| Christianity/Protestant | 11.0% |  |  |
| Islam | 0.3% |  |  |
| Spiritual but not Religious | 3.6% |  |  |
| None | 21.4% |  |  |
| Parents at Home |  |  |  |
| Both Birth Mother and Birth Father | 71.3% |  |  |
| Birth Father and Stepmother | 0.5% |  |  |
| Birth Mother and Stepfather | 7.2% |  |  |
| Birth Father Only | 2.0% |  |  |
| Birth Mother Only | 16.6% |  |  |
| Adopted Parents | 0.8% |  |  |
| Other | 1.5% |  |  |

# **Response Surface Analysis *R* Syntax**

**Model Syntax**

library(RSA)

#### Get Data ####

myData <- read.csv("dataset.csv") #select your data (should be a csv file)

#### Centering ####

midpoint <- 3.5 #enter the midpoint of your predictors (e.g., midpoint <- 2)

#### RSA ####

rsa.model <- RSA(formula = Outcome ~ MoralMindset*AbilityMindset, data=myData, center = midpoint)

summary(rsa.model) #This provides the % of matches & mismatches, coefficients from the polynomial model, and the four RSA

#### plot the response surface in 3-d space ####

plot(rsa.model)
